# The Royal Academy of Medicine and the King of the French

**Published:** 1846-06

**Authors:** 


*The Royal Academy of Medicine and the King of the French.* It is usual for the more important public bodies in France to present to the King, on New Year's Day, an address, to which he generally makes some reply. In accordance with this annual custom, a deputation from the Academy of Medicine was admitted to the royal presence on 1st day of January, when the following address was delivered by the President:—

Sire:—The Royal Academy of Medicine, through our voices, respectfully unites in the concert of gratitude and affection which rises to the throne from every part of France.

Happy to think that it deserves the powerful protection with which your Majesty honors it, the Academy pursues with zeal the course of its useful labors. To endeavor to solve the numerous and varied questions which interest public health; to pursue and forward the progress of science; such is the double mission which is confided to the body which we represent. All the labors, all the desires, of the Academy are directed to one object—the faithful performance of the duties with which it is entrusted; and it dares to hope that its efforts have not remained quite sterile. If it is true that public esteem and approbation have rewarded the endeavors of the Academy, the thanks of its members are due to your Majesty, who deigns to afford to it encouragement, protection, and support.

May the Academy, Sire, present you, as to-day, for many years, the homage of its labors, and the tribute of the gratitude which it feels. This is its most ardent wish as it is that of all France, the tranquility, prosperity, and happiness of which are so intimately connected with the preservation of your valuable existence.

The King answered in the following terms:—

I am gratified and moved by the sentiments expressed in the name of the Academy of Medicine; I appreciate the importance and the utility of your labors, and I am happy to learn that the protection which I so willingly give to the Academy has contributed to increase the merited consideration which it commands. No one better than I can appreciate the services which you render, of which, indeed, I am myself a good example; for in spite of my seventy-two years, I am in good health, as you see. May you gentlemen, long continue to serve humanity and science.

*Ibid.*

